# Setting international standards for patient and parent involvement and engagement in childhood, adolescent and young adult cancer research: A report from a European Collaborative Workshop

**DOI:** 10.1002/cnr2.1523

**Published:** 2021-08-12

**Authors:** Angela Polanco, Reem Al‐Saadi, Suzanne Tugnait, Nicole Scobie, Kathy Pritchard‐Jones

**Affiliations:** ^1^ Bethany's Wish/National Cancer Research Institute (Consumer member) London UK; ^2^ UCL Great Ormond Street Institute of Child Health University College London London UK; ^3^ Great Ormond Street Hospital for Children NHS Foundation Trust London UK; ^4^ Childhood Cancer International Europe Zoe4life Zurich Switzerland

**Keywords:** childhood cancer, Europe, paediatric oncology, parents, patient and public involvement and engagement, strategy

## Abstract

**Background:**

Patient and Public Involvement and Engagement (PPIE) in research, advocates for research conducted ‘with’ not ‘for’ the affected population. In paediatric oncology research, the parents of children, adolescents and young adults affected by cancer are represented by the term ‘public’ in the acronym PPIE. Patients (those with cancer and cancer survivors) are also passionate advocates who drive forward the research priorities of children, adolescents and young adults throughout the entire research process.

**Aims:**

A workshop was held at an international professional meeting in 2019 with the aim to define Patient and Parent Involvement and Engagement (PPIE); capture PPIE activities on a European level; and to explore the role of PPIE in non‐interventional research. A proposed framework for a European PPIE strategy for childhood, adolescent and young adult cancers was also discussed.

**Methods:**

The 60‐minute workshop was attended by health care professionals, researchers, scientists, parents, survivors and charity/support organisations. A presentation to define PPIE, including the difference in terminology for PPIE in the context of childhood, adolescent, and young adult cancers was discussed. Best practice examples from the United Kingdom (UK) helped to demonstrate the positive impact of PPIE in paediatric oncology research. Three breakout groups then explored themes relating to PPIE, namely PPIE priorities, PPIE mapping for Europe, and PPIE in non‐interventional research and data‐linkage.

**Results:**

Disparity in PPIE activities across Europe was evident, with ambiguity surrounding terminology and expected roles for PPIE representatives in paediatric oncology research. A lack of PPIE activity in Eastern Europe correlated with a lack of availability for clinical trials and poorer survival rates for paediatric oncology patients. There was unanimous support for PPIE embedded research in all areas, including in non‐interventional studies.

**Conclusion:**

A European‐level definition of PPIE for paediatric oncology research is needed. Further exploration into the role and responsibilities of patients, parents, and professionals when undertaking PPIE related activities is also recommended. Best practice examples from the UK, France, Germany, The Netherlands and Belgium demonstrated a preliminary evidence base from which a European PPIE strategy framework can be designed, inclusive of the patient and parent voice.

## 1 INTRODUCTION

The language, definitions and goals of Patient and Public Involvement and Engagement (PPIE) varies greatly between stakeholders, cultures, and countries.^1^ Research funders now increasingly recommend and mandate evidence of PPIE in the design, conduct and dissemination plans of funding applications in health research.^1^ However, the definition of PPIE in health research, and acknowledgement of the positive impact that it can have upon the design of a research study and the generalisability of findings; has not yet been widely acknowledged by some health researchers, the general public or by the majority of health care professionals.^2^ Likewise, an understanding of how PPIE (when applied to paediatric oncology research) can be used to drive forward patient‐centred, collaborative projects, is yet to be explored on a European level.^3^

In paediatric oncology research, it is often the parents of children, adolescents or young adults affected by cancer who become actively involved in the prioritisation, design, delivery and dissemination of the research.^3^ They are passionate advocates for their children and are often instrumental in voicing the needs of their child and in raising funds for research where funding may be limited.^3^ In the context of paediatric oncology research, PPIE can be further defined as Patient and Parent Involvement and Engagement (PPIE). In the UK, PPIE embedded research has brought a real‐world, lived‐experience perspective to health research.^4^ It has been proven to not only improve the translatability of research findings, but also increase the likelihood of achieving recruitment targets and recruiting a representative sample into a study.^4^ PPIE methods in research have also helped to disseminate research findings beyond the traditional academic audiences (i.e., in restricted access scientific journals) by sharing the findings directly with the population affected by the disease. To achieve this level of dissemination, the use of lay‐summaries and sharing of findings on trusted social media networks and charity organisations has been recommended.^4^, ^5^

## 2 METHODS

A proposal for a new European paediatric oncology PPIE strategy was presented at an annual international meeting in Prague in 2019 (organised by the European Society for Paediatric Oncology (SIOP Europe) in partnership with Childhood Cancer International (CCI) – Europe. The meeting was by invitation only and featured a dedicated workstream led by CCI‐Europe, for parent and survivor related topics and discussions. As part of the CCI‐Europe programme of events, a workshop hosted by AP (parent), KPJ (SIOPE), RA‐S (scientist), ST (survivor and research professional) and NS (CCI‐Europe) was held with the aim to outline the traditional definition of PPIE and how this term applies to PPIE in the context of childhood, adolescent and young adult cancers. A secondary aim was to outline a framework for PPIE on a European level.

Participants were invited to the workshop via the conference programme and all stakeholders were encouraged to attend. This included parents of children that had a history of cancer, or who currently had cancer, survivors of childhood, adolescent, and young adult cancers, health care professionals, academics and charity or support organisations. The workshop lasted 60 min and featured a presentation to participants with three breakout group sessions. The presentation provided a brief definition of PPIE and then a discussion around what PPIE means in the context of childhood, adolescent, and young adult cancers. An exploration of what PPIE might look like on a European level, alongside exemplar case studies from the UK were then discussed. This was followed by an overview of what the proposed European PPIE strategy hoped to achieve and next steps.

During the breakout groups, participants were invited to form three groups with the following themes for discussion

1. The creation of a European map of countries where PPIE (using the agreed definition) was fully embedded, partially embedded, or not currently featured
2. To discuss how PPIE can be used to help non‐interventional research (e.g., data linkage of large European databases to accurately measure long‐term outcomes of survivors)
3. To identify what PPIE meant to the participants, the barriers to PPIE in their respective countries, and what participants views were surrounding research priorities for improving childhood, adolescent and young adult cancer care and outcomes.

Data were collected from the breakout discussions by the facilitators (AP, NS and R‐AS) and were synthesised by transcription into a workshop report by AP. This was then disseminated to all participants after the conference by email. Contact details were also obtained, with consent, for those individuals that wished to form a working group for the ongoing development of the proposed European PPIE strategy.

## 3 RESULTS

The session was attended by approximately 50 participants. Participants included Paediatric Oncologists, Nurses and Allied Health Professionals, scientists, charity organisations, parents of children, adolescents and young adults that had a history of cancer, and survivors of childhood, adolescent and young adult cancers. A participant poll revealed that >50% of participants did not know the meaning of PPIE or PPIE for the context of childhood, adolescent, and young adult cancer research. Likewise, most participants associated PPIE as being involved in the raising of research funds for new childhood cancer trials. There was little knowledge by participants regarding the number of activities that were considered to be traditional PPIE activities, such as being an author on a scientific research paper or being part of a research steering committee. Over 50% of participants did not know the difference between the levels of PPIE, as defined by the National Institute of Health Research (NIHR) in the UK (participation, involvement and engagement of patients and the public in research).^6^

Despite this, there was evidence of a structured and formalised PPIE network in France, that was also endorsed by the French Government. PPIE representatives in France also had access to tailored PPIE training and were considered to be on the same professional level as scientific researchers. The PPIE structure of France replicated the level of PPIE demonstrated in paediatric oncology research in the United Kingdom. A number of European countries reported examples of parents and survivors having a long‐standing collaborative relationship with paediatric oncology researchers and some with representation on professional committees. A number of charity organisations, run by parents and survivors to support research trials were reported, however they often functioned as a research funding body for targeted research trials for specific tumour types.

Individual breakout sessions revealed a number of reported barriers to achieving PPIE integration as standard in Europe. The disparity in survival rates of children, adolescents and young adults within some areas of Europe and the realisation that some patients still lack basic access to chemotherapy drugs and pain relief options, highlighted the difficult challenge of even setting up and running clinical trials (particularly in Eastern Europe). This, in turn, impacted on the ability to standardise and mandate any form of PPIE in paediatric oncology research in those countries. One participant likened the proposal of mandating PPIE in her country to ‘trying to build a house where there isn't any field to put the foundations’.

**FIGURE 1:**
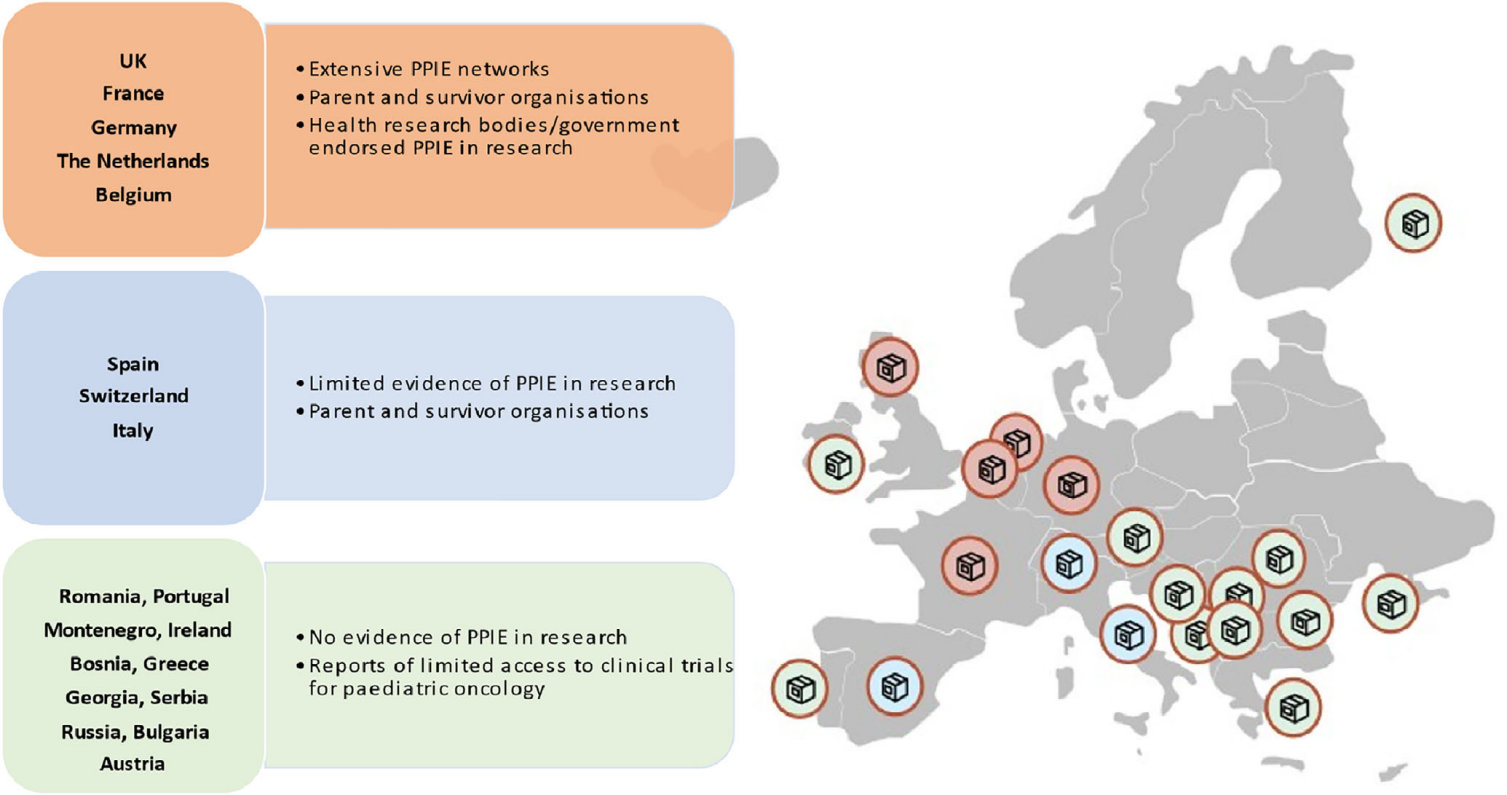
PPIE reported activities in Europe

An initial ‘map’ of PPIE activities in Europe was collated from the breakout discussions between participants. The map represents evidence of PPIE per country (where reported) and the extent of the PPIE activities. Countries where participants reported no knowledge of active clinical trials, PPIE or PPIE, were also captured and illustrated (Figure 1).

Participants from The Netherlands reported that PPIE had been used to help the design of research proposals in paediatric oncology research (although not consistently). There was evidence of PPIE in the reviewing of patient information sheets and publication of results with a lay summary. PPIE had also been used to shape the prioritisation of research questions. Participants from France were able to provide detailed examples of effective and well‐recognised involvement in paediatric oncology research. This included an acknowledgement of the value of PPIE in research by the Ministry of Health. Parents of children with a history of cancer had been involved in and formed part of scientific committees and had also participated in priority setting tasks with the Ministry of Health. France also reported excellent engagement of PPIE representatives for the reviewing of trial documentation and trial design (although not consistently) and had PPIE training resources to ensure that parents and patients were equipped with the knowledge they needed to take part in a variety of PPIE activities. Further examples of PPIE included the writing of lay summaries and the writing of content for a clinical trial website for childhood cancer. The French government was also reported to have funded PPIE activities to assist in priority setting and made calls for new research projects based upon patient and parent contributions. France also maintained close partnerships with The Innovative Therapies for Children with Cancer (ITCC) Consortium and the ACCELERATE early phase trial platform, providing an example of multi‐disciplinary collaboration at the international level.

Participants were then asked to reflect upon their own current paediatric oncology research priorities (by country) and describe how research priorities are currently decided by researchers in their country. Participants revealed that commonly, research trials were based on the health care professionals' research interests rather than patient‐reported priorities and that in their opinion, research should also focus on the long‐term outcomes of children, adolescents and young adults with cancer and not just improving survival rates. The participant reported priority summary is presented in Table 1.

**TABLE 1:** Participant reported research priorities for childhood, adolescent and young adult cancers within Europe

Improving the efficacy of treatment
Increasing the availability and affordability of medicines
Enabling all children to be in a clinical trial
Reducing the toxicity of treatment and late effects
Support for late effects especially psychosocial support for survivors

The integration of PPIE in non‐interventional research proposals, such as systematic reviews and large population‐based studies using pre‐existing data created an active discussion about the importance of international‐level data linkage from population‐based cancer registries and hospital admission data. A need for all countries to have accurate recording of cancer incidence in children, adolescents and young adults, with transparent and robust methods for measurement of long‐term outcomes was voiced by participants.

Participants in the majority, were supportive of the proposal for a standardised European definition of PPIE in paediatric oncology research, and for the development of a PPIE‐focused European strategy. Participants acknowledged the positive impact that PPIE can have upon the design and outcome of a research trial and were keen to share their own experiences of parents and survivors collaborating with researchers in their respective countries. Participants emphasised that in order to develop and implement a European PPIE strategy, support from key organisations such as CCI Europe, SIOP Europe, The European Reference Network, and Joint Action on Rare Cancers platforms was vital. The group also discussed the need for a named point of contact for PPIE within a specific European country to help connect up interested patients/survivors, parents, and researchers. A suggestion for a European‐wide database of PPIE individuals with a linked named contact hosted by either CCI Europe or SIOP Europe would be recommended. Participants then made suggestions for next steps in the development of a European PPIE strategy. These are illustrated in Table 2.

**TABLE 2:** Next planned steps ‐ European PPIE strategy

The development of a PPIE European toolkit with input from key stakeholders
Formation of a PPIE ‘working group’
Funding for priority setting exercise on a European level to find out what is important to children and families in childhood, adolescent and young adult cancers
Lobbying of key European research legislators to ensure that PPIE is a mandatory element of future clinical research projects
Creation of a PPIE database for each country so that professionals can access individuals who are willing to be involved and vice‐versa

## 4 DISCUSSION

Participants of the workshop represented a wide range of stakeholders, providing a unique insight into the multiple definitions of PPIE and PPIE in the context of paediatric oncology research. International examples of PPIE and the potential barriers to the standardisation of PPIE on a European level were openly discussed between participants. This provided key data for the future development of a European PPIE strategy. Despite data only being representative of the participants that attended the workshop, and not the wider academic/professional or public perspective, a notable support for a standardised and collaborative PPIE European strategy for paediatric oncology research was unanimous.

The immediate need of some European countries to first address lower survival rates of children, adolescents and young adults with cancer and the absence of clinical trials in those countries demonstrates the complexities of developing a resource that can be used across Europe.^7^ Similarly, there is a challenge to raise health care professional and researcher awareness of PPIE in paediatric oncology research, particularly the varied scope and depth of PPIE activities. Similarly, the value of PPIE in achieving their recruitment targets.^8^ There is a need to increase awareness about how PPIE can be used to identify research priorities in paediatric oncology research that are patient‐focused rather than researcher‐focused.^8^

The breakout group that discussed PPIE in a wider context (e.g., in large‐scale population‐based trials) emphasised that without an effort to improve international data‐linkage by the paediatric oncology community, the design of early‐phase research trials that aim to increase cure whilst also providing better long‐term quality of life for the patient and family will be more difficult. Participants also agreed that PPIE should be a prominent feature in all types of paediatric oncology research and that there was a need to adopt a standardised definition and collaborative approach to PPIE across Europe.

The concept of PPIE or PPIE in countries where no structure for clinical trials was evident, provoked an active discussion among participants. Some perceived this to be a negative or a barrier to the successful integration of a European PPIE strategy, whereas some viewed this as an opportunity to develop a framework for PPIE embedded research from the grassroots. This would be in contrast to the challenge that research‐established countries might have when trying to adapt existing frameworks and embed PPIE in their current research practices retrospectively. Equally, the work of the European Reference Network and Joint Action on Rare Cancers platform in driving the need for more open clinical trials and a fully inclusive cancer registry for all children with cancer in Europe, could be complemented by the creation of a new European PPIE strategy.^9^ This approach could reinforce the message that PPIE should be an integral part of the research process, not just as an optional extra.

**TABLE 3:** The guiding principles of PPIE (taken from the BRIGHTLIGHT study [12])

Passion – There needs to be passionate people on both sides to keep the movement going and to face challenges, especially on a European level
Preparation – PPIE work needs to be planned, venue, time of day considered, grant applications to have PPIE funds available
Practice – PPIE takes time to be done right, do not expect to get it right the first time and be flexible with your approach and format to PPIE activities
Pounds – Consider reimbursement for time, travel and childcare. PPIE needs to be considered important enough to offer these basic things and not expect parents and survivors to do it for free
Perseverance – Keep going and keep developing initiatives, there will be obstacles and it may not work at first
Post‐it notes – Think of creative and interesting ways to get your data, it is the only way to ensure people will come back and want to be involved again
Patience – Be considerate and compassionate to those taking part and allow for breaks and additional support if needed. Also, allow flexibility in timings and agendas to meet the needs of the PPIE participants

The proposal for a European PPIE strategy for paediatric oncology research was positively received by participants. Existing ‘toolkit’ PPIE resources from the United Kingdom published by The National Cancer Research Institute, Cancer Research UK and the BRIGHTLIGHT study were discussed and illustrated as best practice examples that could be used in the next stage of development.^10^, ^11^, ^12^ The next steps for the European PPIE strategy were proposed and agreed by participants as

- To agree upon a standard definition of PPIE for the context of childhood, adolescent and young adult cancers and disseminate this within the professional and patient communities
- To outline a range of identified PPIE activities and define the expectations/roles of PPIE representatives from the patient/survivor, parent and health care professional perspective
- To draft an educational guide for researchers based on the PPIE and PPIE best practice examples from Europe (e.g., the principles of the BRIGHTLIGHT study, Table 3).^12^
- To undertake a European‐wide online survey to collect data for existing PPIE activities within paediatric oncology research

## 5 CONCLUSION

Patient and Parent Involvement and Engagement (PPIE) throughout the entire paediatric oncology research process is vital to ensure that the focus of any research question, the design of a trial, and the dissemination of results are patient and family centred. The workshop held in 2019 provided convincing evidence that a standardised definition of PPIE (in the context of childhood, adolescent, and young adult cancers) within Europe did not exist. There was evidence of ambiguity surrounding levels of PPIE, PPIE training requirements, and an overall awareness of the value of PPIE for the optimal recruitment and translatable outcomes of research studies.

There were examples of well‐established and long‐standing PPIE models for paediatric oncology research in the UK, The Netherlands and France. However, there is still a disparity in the survival rates of children, adolescents and young adults with cancer in Europe, which presents a difficult challenge for a standardised European PPIE approach. Collaborating with key European stakeholders is recommended for the ongoing development of a European PPIE strategy. A resource to allow collaboration between PPIE interested patients/survivors, parents and professionals is recommended. This resource should also feature in the ongoing development for the European PPIE strategy for paediatric oncology research.

## CONFLICT OF INTEREST

The authors have stated explicitly that there are no conflicts of interest in connection with this article.

## AUTHOR CONTRIBUTIONS

All authors had full access to the data in the study and take responsibility for the integrity of the data and the accuracy of the data analysis. Conceptualization, A.P; Methodology, A.P, K.P‐J, R.A‐S, S.T; Investigation, A.P, K.P‐J; Formal Analysis, A.P, K.P‐J; Resources, S.T, N.S, R.A‐S; Writing‐Original Draft, A.P, K.P‐J, R.A‐S; Writing‐Review & Editing, A.P, K.P‐J, N.S, R.A‐S; Visualization, A.P, K.P‐J, S.T, R.A‐S, N.S.

## ETHICAL STATEMENT

No ethical approval was needed for the workshop, data collection and data analysis. Participants consented for the recording of written notes and analysis of these by the main Author. Consent was provided for those individuals that wished to be informed of the next steps of the project.

## Data Availability

The data that support the findings of this study are available from the corresponding author upon reasonable request.
